# Presence of the knockdown resistance (*kdr*) mutations in the head lice (*Pediculus humanus capitis*) collected from primary school children of Thailand

**DOI:** 10.1371/journal.pntd.0008955

**Published:** 2020-12-16

**Authors:** Narisa Brownell, Sakone Sunantaraporn, Kobpat Phadungsaksawasdi, Nirin Seatamanoch, Switt Kongdachalert, Atchara Phumee, Padet Siriyasatien

**Affiliations:** 1 Department of Parasitology, Faculty of Medicine, Chulalongkorn University, Bangkok, Thailand; 2 Vector Biology and Vector borne Disease Research Unit, Department of Parasitology, Faculty of Medicine, Chulalongkorn University, Bangkok, Thailand; 3 Department of Medical Technology, School of Allied Health Sciences, Walailak University, Nakhon Si Thammarat, Thailand; Hebrew University Hadassah Medical School, ISRAEL

## Abstract

Human head lice are blood-sucking insects causing an infestation in humans called pediculosis capitis. The infestation is more prevalent in the school-aged population. Scalp itching, a common presenting symptom, results in scratching and sleep disturbance. The condition can lead to social stigmatization which can lead to loss of self-esteem. Currently, the mainstay of treatment for pediculosis is chemical insecticides such as permethrin. The extended use of permethrin worldwide leads to growing pediculicide resistance. The aim of this study is to demonstrate the presence of the knockdown resistance (*kdr*) mutation in head lice populations from six different localities of Thailand. A total of 260 head lice samples in this study were collected from 15 provinces in the 6 regions of Thailand. Polymerase chain reaction (PCR) was used to amplify the α subunit of voltage-sensitive sodium channel (*VSSC*) gene, *kdr* mutation (C→T substitution). Restriction fragment length polymorphism (RFLP) patterns and sequencing were used to identify the *kdr* T917I mutation and demonstrated three genotypic forms including homozygous susceptible (SS), heterozygous genotype (RS), and homozygous resistant (RR). Of 260 samples from this study, 156 (60.00%) were SS, 58 (22.31%) were RS, and 46 (17.69%) were RR. The overall frequency of the *kdr* T917I mutation was 0.31. Genotypes frequencies determination using the exact test of Hardy-Weinberg equilibrium found that northern, central, northeastern, southern, and western region of Thailand differed from expectation. The five aforementioned localities had positive inbreeding coefficient value (*F*_*is*_ > 0) which indicated an excess of homozygotes. The nucleotide and amino acid sequences of RS and RR showed T917I and L920F point mutations. In conclusion, this is the first study detecting permethrin resistance among human head lice from Thailand. PCR-RFLP is an easy technique to demonstrate the *kdr* mutation in head louse. The data obtained from this study would increase awareness of increasing of the *kdr* mutation in head louse in Thailand.

## Author summary

Head louse infestation is still a common health problem worldwide. Permethrin is the first line treatment of choice for pediculosis. However, the increase of recurrent cases and treatment failure of pediculosis raises public health concerns about the growing resistance of permethrin among head lice populations. Early determination of the permethrin resistance status is crucial in order to choose the pediculicide of choice appropriately. Our study demonstrated that the PCR-RFLP is a beneficial technique for knockdown resistance detection of T917I point mutation in head lice because it was inexpensive, accurate, and reproducible. Ultimately, our study emphasized that prompt investigation for permethrin resistance should be taken into account in the context of recurrent or treatment failure cases of pediculosis and the alternative chemical pediculicides based on different mechanism of action should be considered under those particular circumstances.

## Introduction

Pediculosis capitis is a well-known infestation of human head louse, *Pediculus humanus capitis* (De Geer). Human head louse is an obligatory hematophagous ectoparasite which complete its entire life cycle on the human scalp by hanging on to the hair. Pediculosis is still very common in children especially the school-aged group. The transmission route is mainly by direct head-to-head contact or on occasion by indirect transmission via fomites, for example sharing contaminated combs, clothing, or towels [1]. Itching is a common symptom of pediculosis capitis due to the release of saliva during head lice sucking its blood meal. The infestation can cause severe pruritus on the scalp of the patient resulting in sleep disturbance and scratching which could be followed by a secondary bacterial infection in the affected area. Pediculosis capitis also interferes with daily life causing social embarrassment, loss of self-confidence, and school absence particularly in school pupils. In addition, *Rickettsia prowazekii*, *Bartonella quintana*, *Borrelia recurrentis*, and *Acinetobacter* spp. have been detected in the head lice but their role as a particular disease transmitter is still inconclusive [2–6].

The prevalence of head lice infestation in Thailand varies from 15.1–88.4% depending on the sampling protocol used for head lice collection [7–9]. The number was particularly high in female pupils especially during the school age. High prevalence of head lice infestation reports has shown partial correlation with lower socioeconomic status [10]. In Thailand, the infestations occurred throughout the country; rural, suburban areas, and even Bangkok, the capital city, but with lower number of cases which could be due to the difference in health concern and socioeconomic status. Most recent reports in the country showed concentration of pediculosis cases in rural compared to urban areas [7–9]. The prevalence of pediculosis predominates in female, a survey reported the prevalence of head lice infestation among female was 50% and 3% in males [10]. Additionally, the survey of the head lice infestations conducted in the North of Thailand found the overall prevalence of 15% and all diagnosed cases were female students [7].

Permethrin, synthetic pyrethrin is a commonly used over-the-counter chemical pediculicide. It is currently recommended as the first-line treatment for pediculosis capitis. Mechanism of action of permethrin as pediculicides acts by binding to voltage-sensitive sodium channels in the nervous system of head louse, causing muscle paralysis and death [11]. Since permethrin has been widely used during the past decades, a large number of treatment failure, recurrent cases, and established evidence of permethrin resistance has been increasingly reported [12–14]. Since then, many pieces of research have been conducted in order to survey the prevalence of permethrin resistance in their countries. The knockdown resistance (*kdr*), due to the three-point mutation (amino acid substitutions on the M815I, T917I, and L920F) can be identified on the α-subunit of voltage sensitive sodium channel (*VSSC*) gene [15–17]. Several reports suggested that M815I and L920F mutations reduce susceptibility to permethrin [18–20], whereas for the T917I mutation, whenever present, whether in alone or combination with L920F mutation, the mutation plays an important role in permethrin resistance and can be used as a molecular biomarker for head lice permethrin or pyrethroid resistance [21,22].

Molecular analysis is one of the most popular techniques used to determine the pyrethroid resistance in insects [19]. Different molecular techniques have been practiced to identify the head lice carrying resistance including quantitative multiplex sequencing [21,23], melting curve analysis genotyping coupled with quantitative PCR fluorescent resonance energy transfer technology (FRET) [24,25], real-time PCR amplification of specific allele (rtPASA), serial invasive signal amplification reaction (SISAR) [11], and restriction fragment length polymorphism (RFLP) [20,26–29].

Recurrent cases of head lice after treatment is still problematic in Thailand. The previous report of the re-infestation rate of head lice after pediculicides treatment among schoolchildren in Bangkok was around 60% [30]. Evidence of permethrin resistance among head lice which could contribute to the increasing of treatment failure rates in Thailand has never been investigated. Previous studies have demonstrated that PCR-RFLP is effective to demonstrate the genotyping of *kdr* T917I by using *Ssp*I restriction enzyme [26–28], therefore, in this study, we aim to investigate the presence of *kdr* mutation in head lice populations collected from schoolchildren of Thailand by using PCR-RFLP and sequencing to screen and confirm the presence of *kdr* mutation in the head louse in Thailand.

## Materials and methods

### Ethic statement

The study was approved and reviewed by the Ethics Committee of the Faculty of Medicine, Chulalongkorn University in Bangkok, Thailand (COA no. 806/2019). The study was explained to each participant and formal consent was filled out by each parent or guardian.

### Head louse samples

A total of 260 head lice DNA samples in this study were obtained from the previous report by Sunantaraporn et al. [5]. The samples were collected from primary school children. The schools are situated at 6 geographical regions, in 15 provinces of Thailand. The DNA samples were stored at -80°C at the Vector Biology and Vector Borne Disease Research Unit, Department of Parasitology, Faculty of Medicine, Chulalongkorn University.

### PCR amplification of the knockdown resistance *(kdr)* fragment

The conventional PCR was performed to amplify the α subunit of voltage sensitive sodium channel (*VSSC*) gene, which contains the *kdr* mutation. Two oligonucleotide primers including kdr-F: 5´AAA-TCG-TGG-CCA-ACG-TTA-AA 3´ and kdr-R: 5´ TGA-ATC-CAT-TCA-CCG-CAT-AA 3´ described by Durand et al. [27] were used for the PCR amplifications. The PCR reaction was performed in the final solution of 25 μl, consisting of 10X PCR buffer, 25 mM MgCl_2_ (Thermo Fisher Scientific, Walthman, MA, USA), 2.5 mM dNTPs (GeneAll Biotech, Seoul, Korea), 10 μM of each primers, 1 U *Taq* DNA polymerase (Thermo Fisher Scientific, Walthman, MA, USA) and 3 μl of the DNA template. The PCR reactions were conducted in a PCR Mastercycler ProS (Eppendorf AG, Hamburg, Germany) the reaction began with initial denaturation at 95°C for 5 min, followed by 35 cycles of denaturation at 95°C for 45 sec, annealing at 55°C for 45 sec, extension at 72°C for 1 min, and final extension at 72°C for 7 min. Positive and negative controls were included in each cycle. The expected PCR amplicons are approximately 332 bp in size. The amplified products were confirmed by electrophoresis in 1.5% agarose gel at 100V for 30 min, and then stained with ethidium bromide. The PCR amplicons were visualized with Quantity one Quantification Analysis Software Version 4.5.2, Gel Doc EQ System (Bio-Rad, Hercules, CA, USA).

### Screening of the *kdr* mutation by using PCR-RFLP

The PCR products were digested in separate reactions with *Ssp*I enzyme to identify the *kdr* T917I mutation (substitution C→T). The RFLP reaction mixture was consisted of approximately 500 ng of PCR products, 10X buffer G, 10U *Ssp*I restriction enzyme (Thermo Fisher Scientific, Walthman, MA, USA), and nuclease-free water to final volume of 10 μl. The reaction was initiated with incubation at 37°C for 90 min, followed by heat inactivation at 65°C for 20 min. The digested products were separated on 10% native polyacrylamide gel electrophoresis at 80V for 90 min using MiniProtein 3 cell (Bio-Rad, Hercules, CA, USA). Gels were stained with ethidium bromide and imaged on a Gel Doc EQ system (Bio-Rad, Hercules, CA, USA). To determine the *kdr* T917I mutation, which is associated with pyrethroid resistance, RFLP was performed by using *Ssp*I restriction enzyme which recognized the AAT|ATT restriction site. When the T917I amino acid substitution occurs, the RFLP pattern should display 2 fragments in RR genotype (261 bp and 71 bp) due to complete digestion and 3 fragments (332 bp, 261 bp, and 71 bp) in RS genotype due to partial digestion. As of the SS genotype (undigested), only one band of 332 bp will be shown [27].

### Nucleotide sequencing

Direct DNA sequencing was performed with the PCR products to demonstrate the presence of the homozygous susceptible (SS), heterozygous (RS), and homozygous resistant (RR) genotype sequences. PCR products were purified using QIAquick PCR purification kit as described by manufacturers (Qiagen, Hilden, Germany). Direct sequencing was performed using the corresponding forward and reverse primers for the *VSSC* gene by a commercial sequencing company (Macrogen, Seoul, South Korea).

To demonstrate the combination of both wild type and mutant sequences in the RS genotype, the PCR products were cloned into the pGEM-T Easy Vector (Promega, Madison, WI, USA) by using a rapid DNA ligation kits (Promega, Madison, WI, USA) following the manufacturer’s instructions. The ligated vectors were assembled for the transformation into competent cells (*Escherichia coli* DH5α), and then the recombinant plasmid DNA were screened using the blue-white colonies selection system. The white colonies suspected to contain the inserted gene were cultured. The plasmid DNA were then extracted using the Invisorb Spin plasmid mini kit (STRATEC molecular GmbH, Berlin, Germany) following the manufacturer’s instructions. Of these at least five clones of the RS genotypes were sent for sequencing by a commercial sequencing company (Macrogen, Seoul, South Korea). The nucleotide sequences were analyzed using BioEdit Squence Alignment Editor Version 7.2.5 [31].

### Statistical analysis of genotype frequencies

Frequency of resistance alleles were calculated by dividing the total number of resistance alleles by total number of all alleles. Genotype frequencies were then tested for the Hardy-Weinberg expectations using a chi-square (χ2) goodness of fit [32]. We used the Wright’s inbreeding coefficient (*F*_*is*_) to test for heterozygous deficiency or excess using the formula,

$$F_{is}=1-\left(Hobs/Hexp\right)$$ (1)

(Hobs; number of heterozygotes observe genotypes, Hexp; number of heterozygotes expected genotypes)

## Results

**Fig 1:**
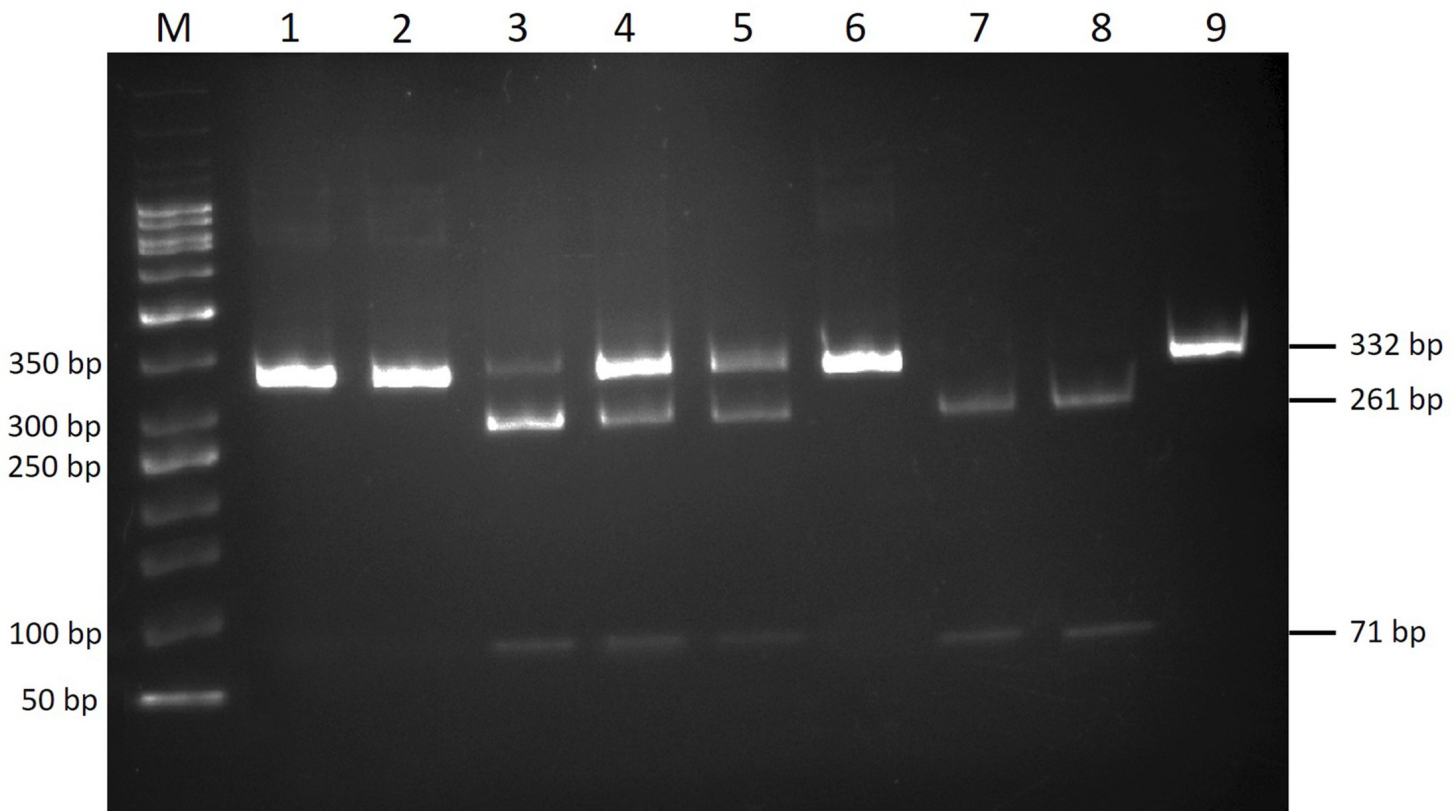
10% native polyacrylamide gel electrophoresis demonstrates the RFLP patterns of *kdr* T917I genotypes. Lane 1 Undigested PCR products, lane 2, 6 and 9 are representative of the homozygous wild type (SS). Lane 3–5 heterozygous genotype (RS) have three bands. Lane 7 and 8 are representative of the homozygous mutation (RR) presented with two bands. Lane M; a 50 bp DNA marker.

PCRs were able to amplify the knockdown resistance (*kdr)* fragment of all head louse samples. The expected RFLP pattern of homozygous susceptible or wild type (SS) is one band of undigested PCR (332 bp), while the heterozygous genotype (RS) is expected to demonstrate 3 different fragments of 332 bp, 261 bp, and 71 bp. For the homozygous resistant or mutant (RR), the product is expected to show distinct fragments of 261 bp and 71 bp (Fig 1).

**Fig 2:**
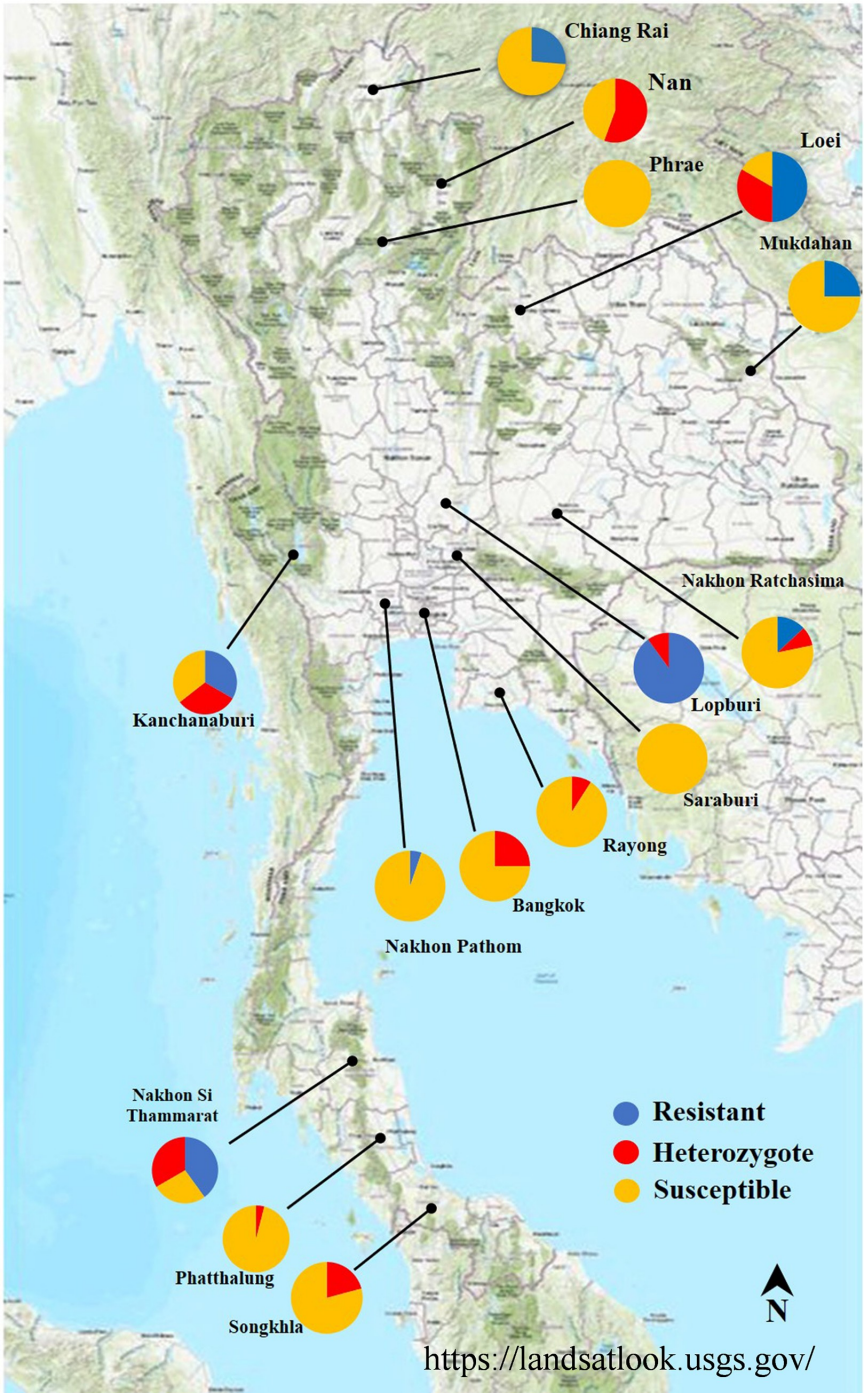
Distribution of *kdr* T917I genotypes in human head lice collected from 6 geographical regions in the 15 provinces of Thailand. (The figure modified from the public domain (https://landsatlook.usgs.gov/)).

Of 260 samples from this study, the SS, RS, and RR were found in 156 (60.00%), 58 (22.31%), and 46 (17.69%) samples respectively. Overall, the frequency of the *kdr* T917I mutation was 0.31 from 15 provinces in the 6 regions of the country. The frequency of *VSSC* gene of all the head louse population in this study ranged between 0.05 and 0.49. The distribution of *kdr* T917I genotype are shown in Fig 2. The Hardy-Weinberg (H-W) model revealed that the distribution of the *kdr* genotype frequencies was not different in five of the six regions which deviated from H-W equilibrium. The eastern region was the only one remaining in the H-W equilibrium. In addition, the *F*_*is*_ of the five regions were higher than 0, suggesting an excess of homozygotes (Table 1).

**Table 1:** Frequency of *kdr* T917I genotype in human head lice collected from different geographical regions of Thailand.

Regions	No. of Samples	*kdr* genotype				HW (χ2)	*F*_*is*_
		RR (%)	RS (%)	SS (%)	Frequency of mutation		
**Northern**	32	5 (15.63)	5 (15.63)	22 (68.75)	0.23	10.20^a^	0.57^b^
**Central**	42	10 (23.81)	2 (4.76)	30 (71.43)	0.26	32.29^a^	0.88 ^b^
**Northeastern**	51	16 (31.37)	10 (19.61)	25 (49.02)	0.41	18.07^a^	0.6 ^b^
**Southern**	79	12 (15.19)	14 (17.72)	53 (67.09)	0.24	20.95 ^a^	0.52 ^b^
**Western**	45	15 (33.33)	14 (31.11)	16 (35.56)	0.49	6.41 ^a^	0.38 ^b^
**Eastern**	11	0 (0.00)	1 (9.09)	10 (90.91)	0.05	0.02	- 0.05
**Total**	260	58 (22.31)	46 (17.69)	156 (60.00)	0.31	89.76 ^a^	0.59 ^b^

RR; Homozygous resistant, RS; Heterozygous genotype, SS; Homozygous susceptible.

a Not in Hardy-Weinberg equilibrium (*P* < 0.05; χ^2^ = 3.84).

b *F*_*is*_ values > 0 indicate homozygous excess, while *F*_*is*_ values < 0 indicate homozygous deficiency

**Fig 3:**
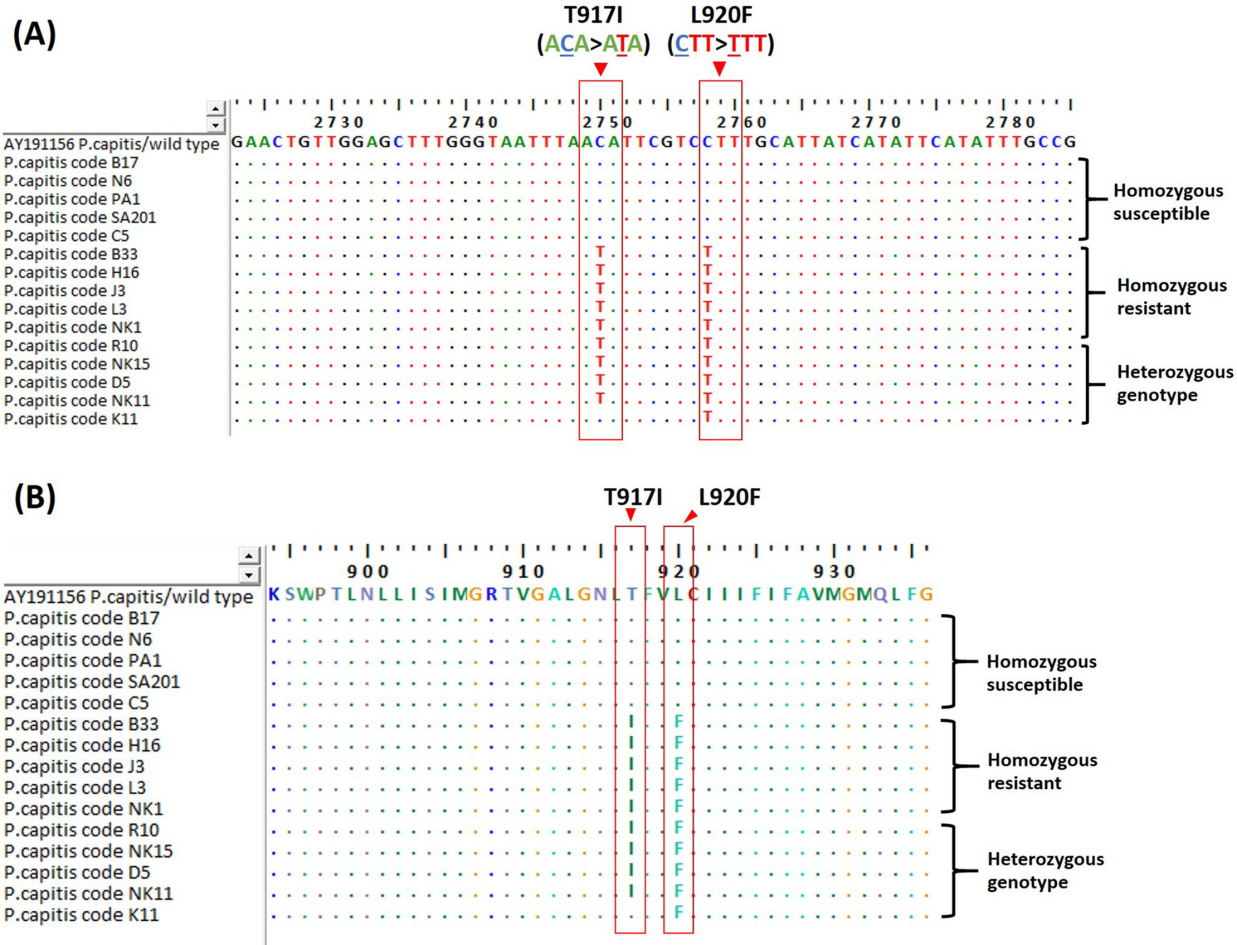
An alignment of nucleotide sequences (A) and amino acid sequences (B) of the α subunit of *VSSC* gene of human head lice collected from Thailand. The sequence showed the position of the *kdr* T917I mutation as well as the position of L920F mutation indicated by a vertical column.

The obtained nucleotide and amino acid sequences were aligned and compared with the published wild-type sequence from GenBank database using the ClustalW function. The insecticide susceptible *Pediculus humanus capitis* (accession no. AY191156), containing the three amino acid substitution of *kdr* mutation sites, was used as the reference sequence. From multiple nucleotide and amino acid sequences alignment, the homozygous susceptible sequences revealed the presence of none substitution on the base of the codon 917, whereas the homozygous resistant sequences showed T917I point mutation due to C→T substitution, leading to Thr (ACA) → Ile (ATA) mutation. Moreover, all homozygous resistant sequences also found the L920F point mutation, which showed the nucleotide substitution of the C→T, leading to Leu (CTT) → Phe (TTT) mutation. The heterozygous sequences were identified in both non-synonymous mutations of T917I and L920F or showed only point mutation on codon 920 (Fig 3).

**Fig 4:**
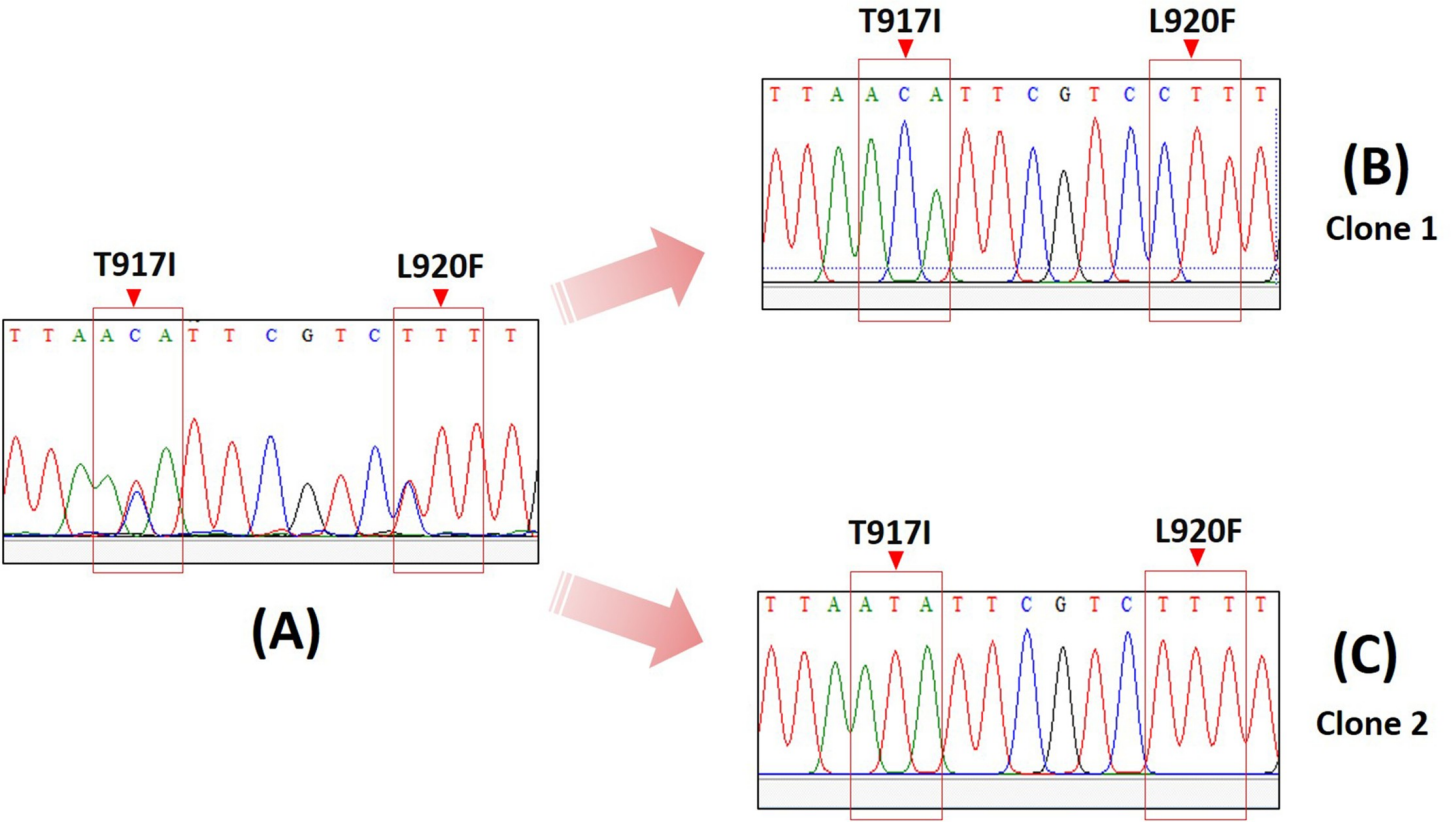
Chromatograms of heterozygous genotype (RS) from direct sequencing (A) equal signal of the nucleotide C and T at T917I and L920F are observed. Chromatograms of clones of the PCR products of the RS clearly demonstrate nucleotide C (B) or nucleotide T (C) signals, showing the *kdr* T917I and L920F of wild type and mutations, respectively.

According to the chromatogram result, the RS genotype sequences obtained from cloning method for sequencing showed different sequences of both wild type (Clone 1) and mutant (Clone 2) at T917I and L920F but the RS genotype sequence from direct sequencing can show the double peaks signal of both C and T at T917I and L920F (Fig 4). The *kdr* sequences were submitted to GenBank under accession numbers MT843902-MT843916.

## Discussion

In our study, we used samples of human head lice collected from primary school children from different provinces in Thailand to screen for knockdown resistance (*kdr)* mutations against pediculicide, permethrin. The overall frequency of the *kdr* mutation is 0.31 in our study which is considerably modest comparing to previous reports of high prevalence of *kdr* mutation in the US, Canada, and Europe [21, 23]. The lower prevalence of *kdr* mutation among head louse populations in Thailand could be due to infrequent usage of pyrethroid in the rural area. The number of infested cases in the rural area tend to be higher than urban area in contrast with their less access to certain pediculicide [7–9]. Consequently, instead of the contemporary scientific drug store pediculicides, traditional methods were the mainstay treatment to eliminate the louse. Folk remedies are based on the use of herb like custard apple leaves, plant-based herbal shampoo, occlusion techniques as well as anti-louse medical devices [33–37]. In urban areas, pediculosis cases are more properly managed with higher number of pediculicides prescription as they are more accessible at either over-the-counter pharmacy or dermatology clinic. The majority of prescribed-pediculicides in our country is permethrin-containing as other pediculicides other than permethrin is not widely available in Thailand.

Regarding overall *kdr* alleles possession among collected head lice, 156 of total 260 lice were homozygous susceptible which is the predominant genotype of these population, 58 lice were homozygous resistant (RR), and 46 lice were heterozygous genotype (RS). Therefore, the lice populations accounted for 40% in either one or two of *kdr* T917I mutation.

Despite the lower prevalence of *kdr* mutation of head lice in our study in Thailand comparing to the other area of the world, one study conducted in 2010 have documented no resistance strain human head lice could be collected from Thailand at that time of survey [18]. The resistance strain of human head lice to pyrethroid was first reported in our present study conducted several years after the aforementioned study. To date, despite a relatively lower tally of resistance in comparison with countries in America or Europe, accumulating evidence suggests that the resistance issue should not be overlooked. The possible explanations of emerging pyrethroid resistance in human head lice could be; 1) Increasing use of insecticides over the past few years, 2) New surveys which cover a larger area with larger sample sizes compared to the previous study, 3) Inadequate or misuse of pediculicides leading to treatment failure, recurrent, or reinfection cases especially in high-prevalence rural area of the country.

To determine pyrethroid resistance in lice, both biochemical and molecular techniques can be applied [27,38]. Molecular approaches were used in this study as they are more widely recognized. We chose PCR-RFLP technique as it was lower in cost, less time consuming, and reproducible. Previous studies have demonstrated that particular point mutations, which was associated with permethrin resistance in the voltage-gated sodium channel of human head lice, was an M815I-T929I-L932F *kdr* mutation [27,29,38,39]. The belief that these three mutations coexist has led to the majority of the recently published papers chose to study T929I mutation in their research experiments to detect *kdr* allele among head lice populations. Yoon et al. [40] established the presence of the T929I and L932F mutation (T917I and L920F, respectively, in head louse amino acid sequence) in permethrin resistance head lice from children in the South Florida. The research is in resemblance to our study, which identified two locations of point mutation.

In order to confirm the reliability of RFLP methods for particular amino acid substitution, we randomly selected samples from three genotypes to perform DNA sequencing. The results of DNA sequencing directly correlated with the pattern of RFLP detected bands in all three genotypes. The chromatogram of the heterozygous genotype simultaneously generated double peaks (two colors) and half the height of the signal intensity at T917I and L920F point mutation. We cloned the PCR products of direct sequencing and randomly picked five representative colonies for further sequencing runs. The chromatogram produced two distinct genomic DNA including wild-type and mutant patterns. The results confirmed the genotypic heterozygosity demonstrating two distinguishing DNA variations. Accordingly, these findings emphasized that PCR-RFLP is a promising technique for knockdown resistance detection in head lice because of its accuracy in identifying the particular point mutations by specific pattern of RFLP bands.

To determine the genotypes frequencies of collected head lice population, we used the exact test of H-W equilibrium and found that five localities except that of Eastern area of Thailand differed from expectation. Additionally, in those five localities also had positive inbreeding coefficient value (*F*_*is*_ > 0) which could be interpreted as homozygous excess. Having excess of homozygotes is an indicator of inbreeding or in fixation of mutant alleles. On the contrary, the first study of pyrethroid resistance in head lice from Honduras by Larkin et al. [20] using RFLP techniques have demonstrated that the majorities of head lice had excess of heterozygotes of *kdr*-type mutations which indicates the active selective pressure among head lice.

In terms of the variation of permethrin resistance in human head lice among the studies, it is believed to mainly stem from the varying prevalence of pediculosis and the frequency of pediculicides use in different countries or continents of the world. However, the overall prevalence has been increasing each year [14,21,23,27,28,41,42].

The *in vitro* mortality bioassay data showed high percentage of agreement between the phenotypic (resistant) and genotypic (homozygous resistance) determinations within the collected South Florida population [40]. The findings from this study emphasized the recessive inheritance of the *kdr* mutation. There was no cross-resistance between malathion and permethrin and those permethrin resistant head lice were still susceptible to malathion. This fortunately supports that the use of malathion and other pediculicides remains an option for treatment.

The first detection of *kdr* resistant head lice in Thailand may be due to repeated episodes of pediculosis especially in elementary school children (with improper prevention) leading to continuous exposure to pediculicides or in some cases, inadequate treatment. The aforementioned explanation could possibly result in permethrin resistant head lice among samples of head lice collected from elementary school children in various area in Thailand. In order to help reduce the unnecessary permethrin or other pediculicides uses, the public health approach of the head lice infestation in the community and school health including educational programs of all related group of people for example parents, school staff, and local health care professionals should be done regularly [37]. In some areas with a high prevalence of pediculosis, the treatment might better lean towards pediculicide-free treatment choices formultiple different reasons as follows; 1) Better safety profile 2) Greater availability in the locals and the advantages of no risk of developing resistant. These treatments include occlusive agents, herbal medicine, and anti-louse devices; heated air, suction, or electronic comb [33–37]. All of which pose no risk of resistance and have the advantages over pediculicides in terms of long term uses. However, for herbal agents, the efficacy and safety are not yet well-established [33,43,44]. Attention has turned towards medical devices rather than medicinal products with less control and regulation. Nonetheless, in some countries, permethrin remains the first line medication for the treatment of pediculosis unless permethrin resistance is suspected in the community [33,43,45].

Our limitation is that the number of collected human head lice in some areas was quite low and might not represent the real situation of pyrethroid resistance in those particular areas. The study was conducted using molecular techniques to demonstrate genotypic resistance in which may not infer the phenotypic level of pyrethroid resistance. Moreover, the collected head louse samples used in this study was of the previous Sunantaraporn et al. study [5] which might not provide the up to date status of head lice resistance to permethrin in Thailand.

In summary, this is the first study in Thailand detecting permethrin resistance in human head lice using molecular method. PCR-RFLP can be used to evaluate the presence of *kdr* mutation in head louse. The *kdr* mutation of head louse in Thailand showed three different genotypes. More number of the collected head lice from thorough locations of Thailand particularly eastern area in future studies are needed to provide a better understanding situation of permethrin resistance among primary school children in Thailand. Further study should focus on the correlation between genotypes and phenotypes of human head lice permethrin resistance.
